# Deducing Hybrid Performance from Parental Metabolic Profiles of Young Primary Roots of Maize by Using a Multivariate Diallel Approach

**DOI:** 10.1371/journal.pone.0085435

**Published:** 2014-01-07

**Authors:** Kristen Feher, Jan Lisec, Lilla Römisch-Margl, Joachim Selbig, Alfons Gierl, Hans-Peter Piepho, Zoran Nikoloski, Lothar Willmitzer

**Affiliations:** 1 Max-Planck Institute of Molecular Plant Physiology, Potsdam-Golm, Germany; 2 Institute for Biochemistry and Biology, University of Potsdam, Potsdam, Germany; 3 Department of Plants Genetics, Technical University München, Freising, Germany; 4 Institute for Crop Science, University of Hohenheim, Stuttgart, Germany; University of Guelph, Canada

## Abstract

Heterosis, the greater vigor of hybrids compared to their parents, has been exploited in maize breeding for more than 100 years to produce ever better performing elite hybrids of increased yield. Despite extensive research, the underlying mechanisms shaping the extent of heterosis are not well understood, rendering the process of selecting an optimal set of parental lines tedious. This study is based on a dataset consisting of 112 metabolite levels in young roots of four parental maize inbred lines and their corresponding twelve hybrids, along with the roots' biomass as a heterotic trait. Because the parental biomass is a poor predictor for hybrid biomass, we established a model framework to deduce the biomass of the hybrid from metabolite profiles of its parental lines. In the proposed framework, the hybrid metabolite levels are expressed relative to the parental levels by incorporating the standard concept of additivity/dominance, which we name the Combined Relative Level (CRL). Our modeling strategy includes a feature selection step on the parental levels which are demonstrated to be predictive of CRL across many hybrid metabolites. We demonstrate that these selected parental metabolites are further predictive of hybrid biomass. Our approach directly employs the diallel structure in a multivariate fashion, whereby we attempt to not only predict macroscopic phenotype (biomass), but also molecular phenotype (metabolite profiles). Therefore, our study provides the first steps for further investigations of the genetic determinants to metabolism and, ultimately, growth. Finally, our success on the small-scale experiments implies a valid strategy for large-scale experiments, where parental metabolite profiles may be used together with profiles of selected hybrids as a training set to predict biomass of all possible hybrids.

## Introduction

Maize is one of the most important crop plants and its total annual production of 883 Mt, as of 2011, exceeds the production of other major crops, like rice or wheat, by 20% (http://faostat.fao.org). In addition to its agronomic importance, maize has been a model organism for biological research for nearly a century. The integration of scientific knowledge into the breeding practice resulted in a nearly linearly increasing average yield in maize production from about 1.9 to 5.2 t/ha over the last 40 years. Besides improvements in cultural practices, like irrigation and fertilization, a constant development of superior cultivars and the exploitation of the heterosis phenomenon contributed to this success, with estimated genetic contribution to yield increase due to hybrid breeding of 50–60% [1].

Heterosis describes the phenomenon that hybrids exhibit superior performance relative to parental phenotypes [2]. In an outbreeding crop, like maize, absolute heterosis of more than 100% can be observed relative to the better of the inbred parents for some traits [3], but the extent of heterosis generally depends highly on the parental genetic backgrounds and the environmental conditions [4], [5]. Breeding programs try to identify the most promising hybrids among various parental combinations. As this becomes labor intensive for higher numbers of parental lines, prediction of hybrid performance (HP) based on parental traits has long been under scientific investigation [6]. Traditionally, phenotypic measures like General and Specific Combining Ability (GCA and SCA) were obtained for this purpose. These measures estimate HP based on the performance of Test Crosses (TC) of the parents with other lines and was originally conducted by modeling univariate traits with linear models given parental labels (see [7], [8] for modern examples), but can be expanded to model vectors of traits as demonstrated in [9].

Parental labels alone are often not sufficiently predictive of HP. Utilizing technological advances, various genetic markers have been extensively tested as new or refined additional predictors for HP using various mathematical approaches, including: linear regression (LR), best linear unbiased predictors (BLUP), support vector regression (SVR), and Bayes approaches [10]–[14]. The achieved predictive power for a given trait (e.g., grain yield) varies greatly (for a nice review, see [15]). Riedelsheimer [19] is a recent example whereby the hybrid biomass and bioenergy related traits are combined into a single GCA value for the corresponding parents, and the GCA value is then predicted using ‘omics’ data measured on the parents.

In an extensive *in situ* experiment [16] to quantitatively investigate major influencing factors on prediction accuracy, inter-population structure and type of validation group were shown to be the main contributors to the observed variance with obtained prediction accuracies varying from 0.65 to 0.95 and measured as correlation *r* between predicted and observed trait value. In short, it is less difficult to achieve a good prediction performance for (i) hybrids produced from divergent parental populations, *i.e.*, where parental lines are genetically more unrelated, compared to convergent parental populations, and (ii) hybrids for which TCs (half-siblings) from one or both parents are evaluated within the training set compared to hybrids where no such lines were included. Marker density, in contrast, had only a minor effect on prediction accuracy, setting a limit to the usefulness of additional genetic markers in a model.

Important agronomic traits are typically highly polygenic, and are under the control of a large number of quantitative trait loci (QTL) with small effects–a hard nut to crack with QTL-based marker-assisted selection methods. Additionally, the identity, the genetic function and interaction of specific genes associated with heterosis of different traits is mostly unknown. More detailed information may be obtained by inspection of other molecular traits, like transcript or metabolite levels, which integrate genetic and environmental influences [17], [18]. The first complementary testing of large-scale genomic and metabolite data to predict important agronomical traits in hybrid maize test-crosses concluded that the prediction accuracies of heterotic traits in adult maize plants using metabolite profiles of the young leaves were only slightly lower than with Small Nucleotide Polymorphisms (SNPs), although metabolites represent approximately 300 times smaller number of variables compared to SNPs [19].

Heterosis is typically investigated in adult hybrid plants, however, this phenomenon already manifests during the very early stages of seedling development [20]. The development of the primary root as first organ allows the comprehensive analysis of maize seedlings prior to the shoot emergence a few days after germination since a number of heterotic traits were described on the macroscopic (morphological and histological) [20] as well as on molecular (transcriptome and proteome) [21], [22] levels during early postembryonic development. Although, the primary root system contributes little to the season-long maintenance of the corn plant, it helps sustain seedling development by virtue of water uptake, and is important for early vigor of the maize seedlings [23]. In order to enable tight control over environmental parameters for plant growth and metabolite data collection, primary root was used as model system in this study.

Previously, we reported metabolite and biomass data of primary roots obtained by full diallel mating design of four European maize lines (two dent and two flint lines). The results led us infer that hybrids show optimized metabolic flux configurations with respect to biomass optimization [24]. It is reasonable to assume that the metabolic levels leading to optimized metabolic flux configurations are constrained by the genetic possibilities inherent in the particular parental combination (along with the 'standard' biochemical constraints) and, therefore, that parental metabolite levels may allow the prediction of complex heterotic traits, e.g., hybrid primary root biomass. This question was already investigated with some success in a large Arabidopsis data set [25], [26] where it was shown that feature selection, *i.e.*, a filtering process retaining only a minimal set of markers containing the relevant information, was a critical step to improve HP prediction and, further, that variable importance in the projection (VIP) can be used for this purpose [26].

There are many frameworks for prediction of macroscopic phenotype directly from the parents. To our knowledge, prediction of molecular phenotype such as hybrid metabolic profiles has not been previously attempted, although this could enhance prediction of macroscopic phenotype. In this work, we aim to investigate the prediction value of parental metabolite profiles for hybrid metabolite levels and biomass production during the very early stage of maize seedling development. Here, we present methods to (1) transform hybrid metabolite levels relative to parental levels by using standard concepts of additivity and dominance, thereby implicitly retaining the diallel structure, (2) predict hybrid metabolite phenotype given parental metabolite profiles and (3) use the results of (2) as a feature selection method to predict hybrid biomass directly from parental profiles. We find a subset of parental metabolites which are not only predictive of hybrid molecular phenotype but also of biomass.

## Methods

### Plant Material and Growth Conditions

The maize inbred lines UH002, UH005, UH250 and UH301 as well as their 12 hybrid combinations were generated in the nursery of the University of Hohenheim in the summer season of 2003. Seeds were surface sterilized, thoroughly rinsed in twice distilled water, transferred on moistened filter paper (193×290 mm Grade 603 N, Munktell&Filtrak, Bärenstein, Germany) which was rolled up with 10 seeds of a genotype per filter paper and germinated in a phytochamber (Versatile Environmental Test Chamber, MLR-350, Sanyo, Japan) at 26°C, with a 16 h light and 8 h dark cycle [20]. For further analyses, the 3.5-day-old roots were excised with a razor blade, the roots growing on the same filter paper were pooled, weighted, snap frozen in liquid nitrogen and stored at −70°C. This procedure was repeated six times per genotype leading to six biological replicates. Altogether six times ten kernels of 12 hybrid and 4 inbred genotypes were in randomized order independently germinated and harvested. For each sample the average biomass (fresh weight of 10 pooled primary roots) was calculated, these values represent the primary root biomass in the very early stage of maize seedling development. Frozen samples were randomly grinded in 2.0 ml round bottom micro-vials (Eppendorf, Germany) with prewashed 0.25 inch steel balls in a mixer mill (Retsch, Haan, Germany). Per sample 100 mg of frozen homogenized pooled root material was subjected to subsequent sample extraction.

Root material was preferred over analyzing kernels to account for heterosis effects during seed formation (accumulation of storage compounds) as well as seedling establishment (storage compound utilization and environmental influences).

### Metabolomics Analyses and Data Normalization

A targeted analysis [27] evaluating the levels of 112 distinct metabolites was conducted for six biological replicates of each individual genotype following the procedure outlined in [28] and modified as described in [29] with respect to the extraction mixture (MeOH:MTBE:H_2_O instead of MeOH:CHCl_3_:H_2_O). The 112 metabolites are a subset of the extractable polar fraction of metabolites which are accessible by gas-chromatography-mass spectrometry (GC-MS) and where selected after manual inspection of several chromatograms. Sixty nine metabolites were identified by comparison with the Golm Metabolome Database [30] as a reference based on Retention Index and spectra similarity. For 19 of the remaining 43 unidentified metabolites we could assign a putative chemical class (aa: amino acid, acid: organic acid, cho: sugar, chop: sugar phosphate) according to selective masses from the spectra. All samples were measured in completely randomized order in three consecutive batches (measurement days).

Metabolite intensities were log_10_-transformed to better resemble a normal distribution. A two-way analysis of variance (ANOVA) was applied using genotype and sample batch as factors. Systematic differences due to the latter factor were thus removed. Values with studentized residues larger than four were eliminated. In a further normalization step, we corrected for differences in metabolite levels due to variation in initial sample amount. Here, we calculated a correction factor for each sample as the ratio of its median peak height (*i.e.* metabolite level) and the median peak height for all replicates of the similar genotype. By dividing each sample with its correction factor, we scaled biological replicates to a similar median peak size.

### Notation

Let 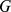 be the set of parental genotypes with 
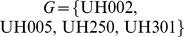
, and 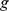 denote a member of 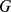, *i.e.*, 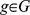. A hybrid genotype is denoted by 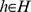, where 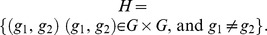 In total, there are 12 different hybrid and 4 different parental genotypes.

Let 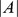 denote the cardinality of a given set 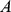.

Let 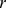 denote the number of available replicates for each measurement. The 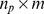 matrix 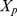 gathers the profiles of 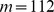 metabolites from 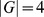 parents and 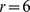 replicates, thus, 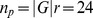. The matrix 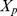 will be referred to as the data matrix of parental metabolic profiles. Analogously, the 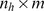 data matrix 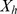 gathers the hybrid metabolic profiles, where 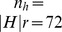. Columns of 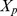 and 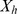, corresponding to metabolites, are mean-centered and scaled to unit variance.

Let 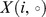 denote the 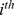 row of a matrix 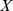, and 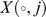 its 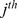 column.

We next construct an 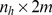 matrix 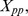 where each row represents a hybrid as a concatenation of two parental profiles 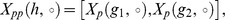 also mean-centered and scaled to unit variance.

Notation regarding replicates is suppressed and it is always implied that a group of replicates is meant when a genotype is discussed, unless otherwise stated.

### Problem Setting

Every 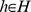 can be represented on 4 levels:

- A: the labels *g*_1_ and *g*_2_ of its parents
- B: the combined metabolic profiles of both of its parents
- C: its own metabolic profiles
- D: its biomass.

In practical terms (e.g., a breeding program), it is desirable to predict macroscopic quantities such as D given an easily obtainable quantity describing its parents. Efforts have been made for decades to predict D given A by using linear models, culminating in the Bayesian formulation found in [7].

A black-box approach would be to predict D given B, however, level C is skipped which potentially has predictive information. Levels B and C could also stand for other types of molecular data, such as transcript or protein levels. Additionally, there would be the need to select features of B to gain biological insight or develop a small number of predictive biomarkers for use.

Here, we aim to predict C given B. In general, it is hard to predict one profile given another, hence we apply the following simplification: if 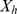 is the matrix of profiles corresponding to C, and 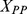 corresponds to B, we predict 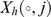 given 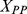 for each 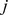. The trade-off of this simplification is that the individual metabolites in the hybrid profiles are treated as if they are independent of each other, which is clearly not true for each metabolite.

The output of the parallel prediction problems (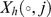 given 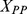) is aggregated and some parental metabolites (labelled as either maternal or paternal) show an overall higher predictive power of 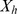
than others. Therefore, we use this as a biologically-motivated feature-selection method and find that these parental metabolites are also predictive of biomass, *i.e.*, allow to predict D given B.

### Problem Formulation

A new 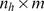 matrix 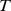 is first constructed, quantitatively capturing the concept of additivity and dominance, by comparing the levels of each metabolite in 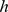 to those in the respective parents. As a result, hybrid metabolite levels are expressed relative to the corresponding parental levels and not to a common reference (zero). This captures the genetic constraints imposed by the parents, and is achieved by using moderated t-statistic [31], as detailed below.

For every metabolite 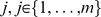 and every hybrid 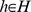, we consider the following two null hypotheses for i = 1 (maternal) and i = 2 (paternal):
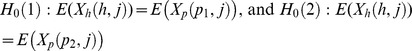



Because for each 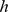, there is a multiple testing situation, moderated t-statistics are calculated over 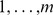 for each 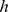.

Using this approach, each metabolite 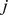 within each hybrid 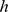 is given a label 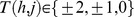 specified by:



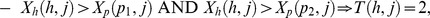





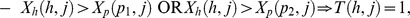




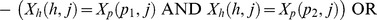
 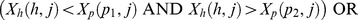 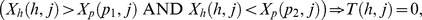




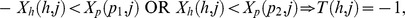





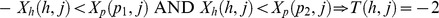
where, for succinctness, the notation for expectation 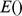 is neglected for all 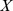 and ±2 corresponds to positive/negative overdominance, ±1 corresponds to positive/negative dominance and 0 corresponds to additivity, respectively. Therefore, in the alternative formulation, the problem is that of classifying the parental matrix 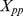 according to:
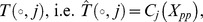
where 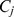 is a classifier to estimate 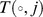 given the parental matrix 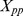 as input.

Let 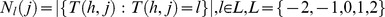 denote the number of genotypes with the corresponding class label in metabolite 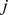. Hybrid metabolites are then filtered so that only those with reasonably balanced classes are predicted by assigning each hybrid metabolite 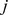 a weight 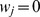 where:


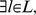
 such that 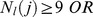



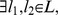
 such that 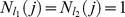 and 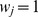, otherwise. For two of the remaining metabolites where 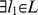, such that 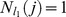, we removed only the rows of the corresponding genotype.

In a classification problem, given a data set of points, belonging to one of at least two classes, it is required to determine a function of the features, specifying the points, to infer the class labels. Depending on the properties and constraints the function should satisfy, there are several approaches available, and a thorough overview can be found in [32]. Here, the class labels are given by the CLR, and the features are the parental metabolites. To infer the class labels, we employ five classification methods: support vector machines (SVM) VAPNIK, linear discriminant analysis (LDA) [32], random forests (RF) [33], RF preceded by a partial-least-squares dimension reduction step (PLS-RF) [34] and LDA preceded by a partial-least-squares dimension reduction step (PLS-LDA) [34]. Selecting a classification method 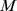 (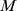: SVM, LDA, PLS-LDA, PLS-RF, RF) for a problem based on lowest class error rate of an individual method can give an 'optimistic bias' [35]. Therefore, we used the following strategy to obtain those metabolites which are well classified regardless of the classification method employed (available from the Bioconductor package CMA [36]):

For each 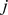 with 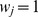:

1. Construct a new 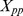, after permuting the rows 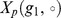 and 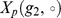, corresponding to each 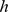.
2. Split 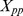 into 3 groups of samples for 3-fold cross validation (sampling balanced across classes)
3. Construct the classifier 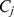 3 times using each group once as the test set, and apply different classification methods to estimate either the observed labels 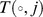 or a permuted version 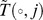 thereof.
4. Report the median misclassification rate 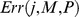 for method 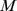 and 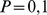 for 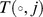 and 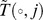 respectively.
5. Repeat steps one to four 25 times.
6. For each method, report the median misclassification rate 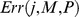 over the 25 replicates. Select the two methods 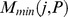 with 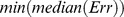 in both 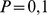.
7. Define 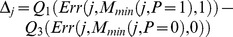, where 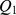 and 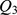 denote the first and third quartiles, respectively, of 25 median errors 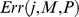. First and 3rd quartiles are used to be stricter than comparing medians.

If 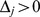, then 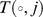 is considered to be predictable using 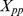. For these metabolites, it is now desired to select the features of 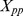 which are most predictive of 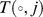. To do so, ranked feature weights of SVM is used (regardless of performance compared to other methods, as this remains unknown), *i.e.,* for each 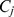, there is a 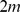-vector of parental metabolite feature weight ranks 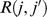, where 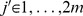 and indexes the parental metabolites. Ranks are used to avoid the problem of feature weights being on different scales for each 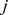, and to avoid the problem of threshold selection.

To summarize the combined performance of all hybrid metabolite classifiers, the parental metabolites 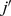 are ranked based on the median of 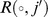, *i.e.,* their importance in predicting each 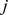. Parental metabolites with a low 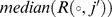 are often important in predicting 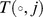 and conversely metabolites with a high median, are not very often important in predicting 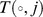.

### Validation of Selected Parental Metabolites Using Hybrid Fresh Weight

To test if the informative parental metabolites, which are low ranked in hybrid metabolite prediction, are also predictive of biomass (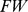), we form a final ranking for parental metabolites 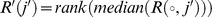.

We form a biomass predictor 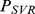 using support vector regression on 60% of the samples as a training set and measuring performance calculating the Pearson correlation between the predicted (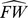) and actual biomass values.
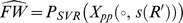



To determine the subset of columns (metabolites) of 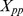 selected we define 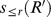 as being 5 randomly selected metabolites out of those with 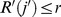, and 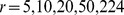, and 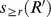 with 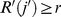 and 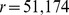. This is compared to 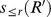 for 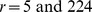 with the 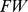 values block permuted, *i.e.* biological replicates for each 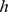 remain together. For each 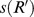, 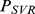 is constructed 500 times, each time with rows randomly assigned in 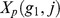 and 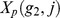, as well as 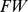 replicates also being randomly assigned.

A schematic representation of our analysis pipeline can be found in Figure S6.

## Results

### Description of the Experimental Setup and Conceptual Framework

We used four European parents, two of each from the flint (UH002 and UH005) and the dent (UH250 and UH301) pool, and all their reciprocal hybrids. The full experimental design is displayed in Figure S1 B and was also previously described [24].

Based on our earlier observation that biomass was correlated to the deviation from a set of optimal metabolic levels, we concluded that in order to complete a targeted breeding approach, it is crucial to establish the link between parental and hybrid profiles. While it is easy to select promising parental lines (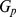) and measure their metabolic profiles (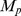), we set out to devise a method to infer from 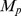 the hybrid profile (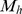), or a derived version (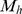) thereof, retaining sufficient information to predict hybrid biomass (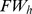) ultimately based on parental traits alone (Figure S1 A, Materials and Methods).

As metabolism is sensitive to changing environmental conditions [37], our experiment was designed to keep environmental influences at minimum. Therefore, we performed our study on the germinating root system in maize, where heterosis was previously shown to occur in a highly controlled setup [20]. Six biological replicate samples, each containing 10 pooled roots, were analyzed by gas-chromatography time-of-flight mass-spectrometry (GC-TOF-MS) to obtain the metabolic profiles comprising the levels of 112 metabolites [24].

We initially tested if biomass can be predicted based solely on knowledge of the parental genotype and biomass, utilizing a Bayesian framework [7] to estimate the posterior densities of the inherent effects. However, hybrid outcome is essentially arbitrary in the absence of further information, and there is no power for further generalization (e.g., parent X improves hybrid biomass independent of the other parental genotype). Therefore, to gain a deeper insight, we next investigated the connections between parental and hybrid metabolite profiles and average root´s biomass.

### Re-encoding the Hybrid Metabolite Profiles According to Individual Heterosis Mode of Action

Hybrid metabolic levels depend on parental levels, albeit in an unknown way. To investigate the connection, we do not work with absolute hybrid metabolic levels but rather we transformed them to relative values with respect to the corresponding parents. However, here each hybrid metabolite is compared to two separate quantities, namely the corresponding maternal and paternal metabolite levels, and a decision must be made on how to combine the parental levels. Representing the parental levels by the mean may not suffice, because the separation between the parental levels is lost and this is essential information about the diallel structure. Instead, we define the Combined Relative Level (CRL) by applying the concept of additivity/dominance/over-dominance to each metabolite. If the hybrid level is significantly greater/smaller than both respective parental levels, then CRL is +/−2. If it is significantly greater/small than just one parent, CRL is +/−1. When it is indistinguishable from both parents or greater than one and smaller than the other, CRL is 0 (cf. Methods). While information about the diallel is retained through the consideration of the separation of each parental combination in the calculation of the CRL, it is evident that the magnitude of the hybrid shift is lost.

Our aim was to examine whether certain regions of parental metabolite space favor certain shift directions, as a consequence of common genetic and biochemical constraints. However, while we did the classification individually per metabolite, hybrid metabolite levels are likely to be the outcome of complex combinatorial patterns of multiple parental metabolites levels [38]. The corresponding parental metabolite levels of metabolite 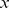 may even be less influential for the hybrid outcome of 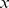 than the parental levels of metabolites 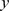 and 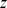, which potentially allows the prediction of hybrid outcome based on a reduced set of parental metabolite levels.

### Predicting the Hybrid Class Label Profile

We asked for every hybrid metabolite which of the parental metabolites is predictive of the hybrid class labels based on their levels. The parental input matrix 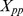 is constructed as a concatenation of maternal (m) and paternal (p) profiles (*cf.* Methods) and, therefore, contains every metabolite twice (*e.g.* alanine_m_ and alanine_p_).

**Figure 1:**
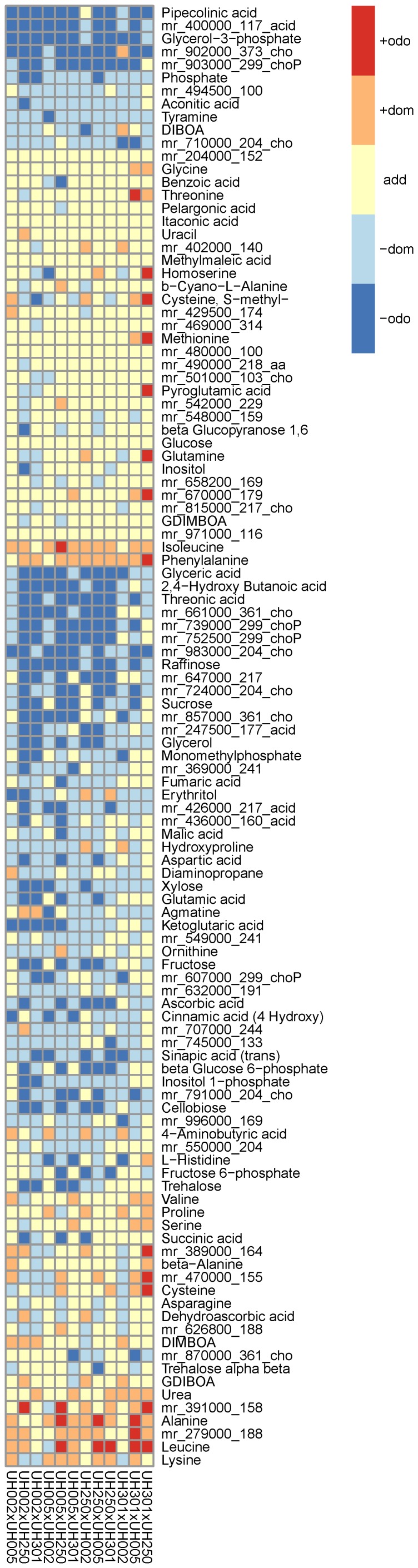
Hybrid class label matrix. The hybrid class label matrix is established using moderated t-statistics (cf. Methods). It shows the observed metabolite heterosis mode of action in all hybrids. Metabolites with unbalanced class labels (e.g. predominantly showing similar class, upper 53 rows) were excluded before conducting classification methods. Various classification methods were used on parental metabolite data to investigate which parental metabolites allow to predict the observed classes within hybrids.

Before classification, hybrid metabolites which show the same class label at least nine times (out of 12 combinations) were removed. This is necessary to avoid an overly unbalanced set of class labels, narrowing down the profiles to 69 metabolites (Figure 1). The threshold of nine was chosen based on a visual inspection of class label balance distribution. We then tested several classification methods (support vector machines (SVM), linear discriminant analysis (LDA), random forests (RF) and combinations of partial least squares (PLS) with the previous: PLS-LDA and PLS-RF, cf. Methods for details) which are ideally evaluated against an independent test data set to avoid choosing the ‘best’ method. Because such a test data set is currently not available, we compared our results to classification with permuted class labels using 3-fold cross-validation on both permuted and original datasets, each time with 25 replicates. For the 54 metabolites where the minimum original median error from one of the classification methods was lower than the minimum permuted median error we considered parental profiles to have predictive power for the hybrid class label. The median miss-classification frequency for the SVM method, which often has a low CV error, is shown in Figure S2.

### Identifying the most Influential Parental Metabolites

**Figure 2:**
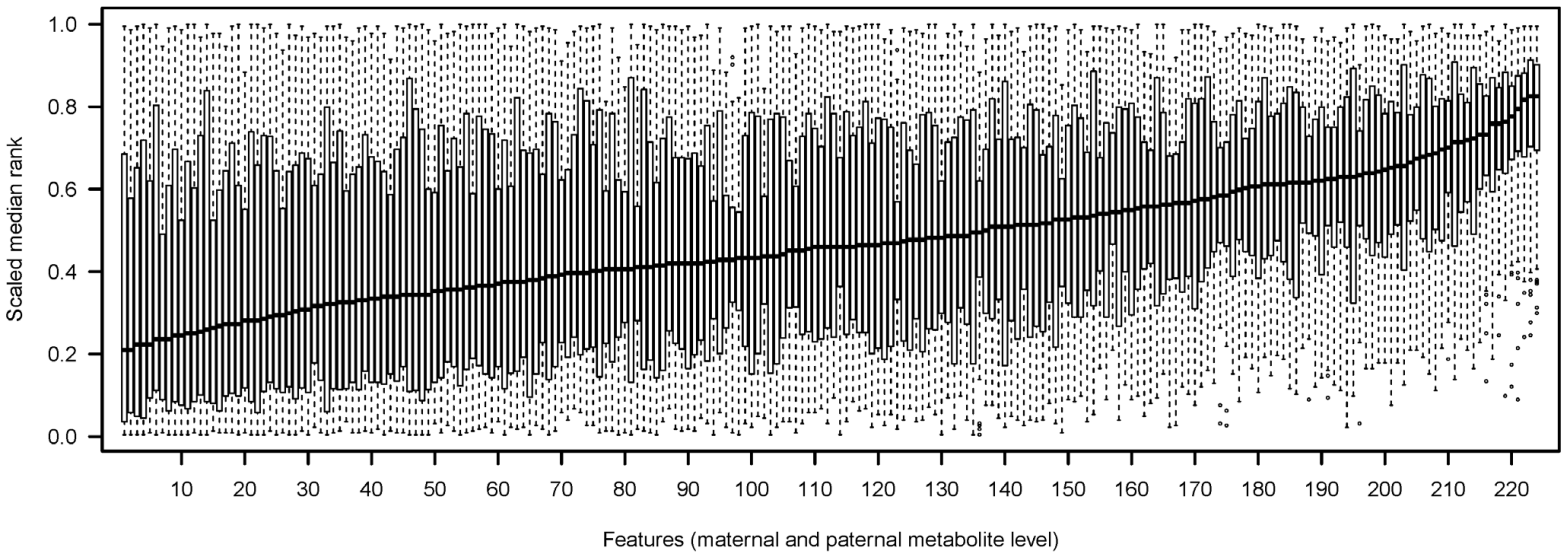
Ranking of the parental features. Parental metabolite levels (224 features in total) are used to predict the observed class labels of 54 hybrid metabolites. In each prediction model all features can be ranked according to their weights. The ranks are scaled between 0 and 1 by dividing by the total feature number. The scaled median rank distribution of a feature, *i.e.* the individual boxes in the plot, then gives an estimate regarding the importance of the absolute parental level of the respective metabolite on the heterosis pattern of all hybrid metabolites.

The next question to address was which parent metabolites are influential in the prediction of hybrid class labels which requires a feature selection procedure. As SVM performs well overall, we decided to use the embedded feature selection method, *i.e.*, recursive feature selection. However, the feature weights appeared to be on different scales for each hybrid metabolite, and, furthermore, there was the additional problem of choosing an appropriate feature weight threshold. To circumvent these challenges, the feature weight rankings were used, where a low rank corresponds to high feature weight or variable importance. The median ranks over all 54 hybrid metabolite class label predictions were scaled between 0 and 1, allowing the identification of parental metabolites with global importance, rather than individually choosing and interpreting a set of features for each metabolite. The rank distribution within each feature (parental metabolite) over all predicted hybrid metabolites is shown in Figure 2, where metabolites are sorted by their median rank.

It can be seen that features with low median rank are also highly skewed to the left, meaning they are low ranked more often than high ranked. At the other end, there are features which are never of low rank. This implies that there is a set of parental metabolites which may be implicated in the outcome of the discretized hybrid metabolite profiles (CLR), *i.e.* they are informative not only for the hybrid heterosis mode of action for the respective metabolite itself but for several up to many metabolites.

To assess how robust our feature ranking would be if not all genotypes are included in the modeling step we performed a leave-one-out (LOO) approach excluding all replicates of a specific hybrid. This is important for a later application in breeding where we would like to make predictions on hybrid traits based on their parental properties without measuring the hybrid itself. While it is obvious that a LOO strategy is less strict compared to an independent test set, we found the feature ranking to be very stable (Figure S3).

### Predicting Hybrid Root’s Biomass from Parental Metabolite Profiles

We have been able to predict the CRL class labels of each hybrid metabolite individually given the parental profiles, and some parental metabolites are overall more influential than others. Furthermore, this ranking does not appear to be dominated by any genotype in particular, given that the feature ranking is stable using a LOO approach.

**Figure 3:**
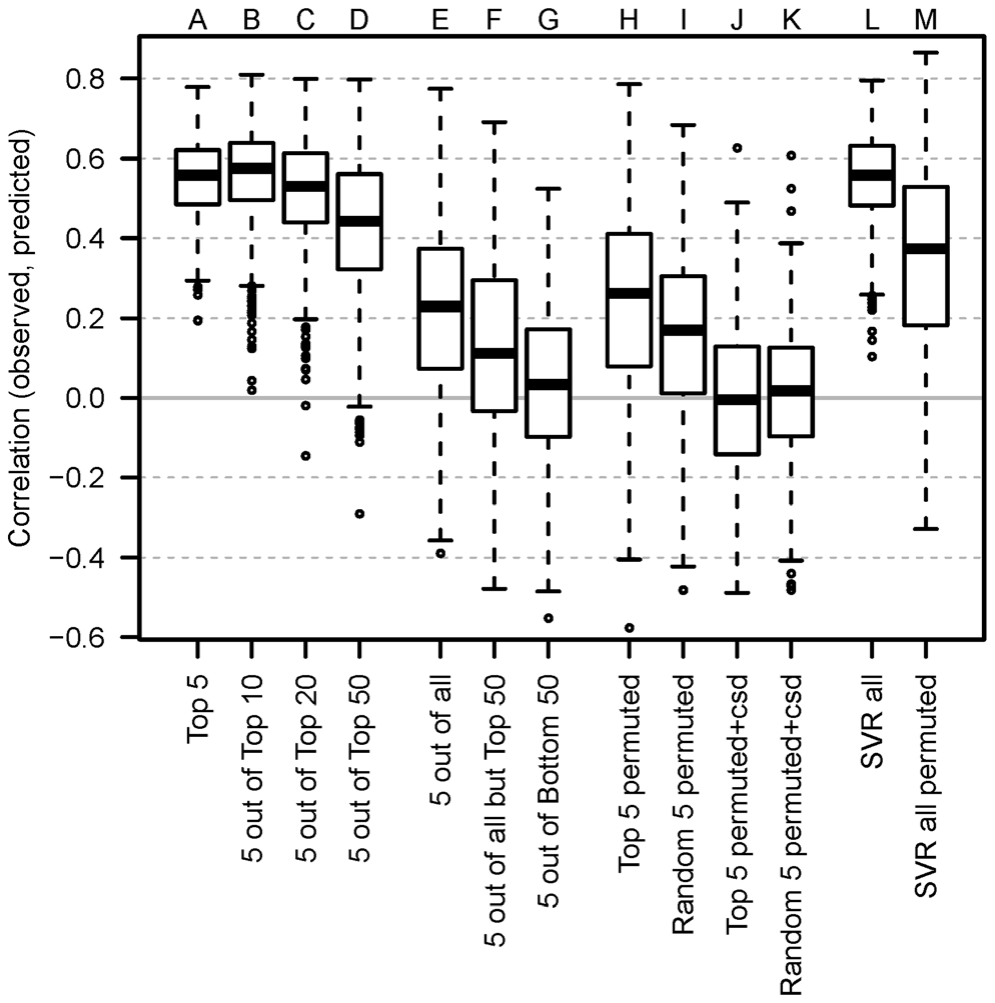
Prediction evaluation. The correlation between observed and predicted HP in models incorporating 5 metabolites. The metabolites have been selected based on a previous ranking (cf. Figure 2). It can be seen that good prediction accuracies are only obtained for high ranked metabolites and not for low ranked metabolites, which perform comparable to permuted data sets. Metabolite input matrices *X_pp_* were established as described in Methods.

It would be of practical use to predict the biomass of primary roots in the progeny given parental profiles, and thus we now investigate whether the feature ranking can also be used for feature selection. We predict the biomass given the parental profiles using support vector regression (SVR), and as a baseline, we use all metabolites, with prediction quality measured by correlation between actual and predicted biomass values (Figure 3, Box L). Comparing the prediction quality to that of permuted biomass values, the parental profiles are indeed predictive of average fresh weight (Figure 3, Box M).

We would like to know whether all features are necessary for prediction, or whether a small number of features may achieve comparable predictive power. We find that using only the top 5 ranked features in the SVR gives comparable results to using all features (Figure 3, Box A). We would like to know how many of the top ranked features are equally good predictors. To this end, we randomly select 5 out of the top 10, 20 and 50. The top 5, 10 and 20 features have comparable prediction quality (Figure 3, Box B and C), and there is a decrease in prediction quality by using the top 50 (Figure 3, Box D). Note that the number of features used in the SVR remains fixed at 5, as a higher number of features was found to improve prediction quality for the top 10 and top 20. Furthermore, prediction quality progressively decreases as more bottom ranked metabolites are included in the SVR (Figure 3, Boxes E, F and G).

The results of the top 5 and random 5 can be compared to predictions of permuted biomass (Figure 3, Boxes H and I), and the top 5 features are also predictive of biomass, while it is not true in general that randomly selected features are predictive. Note that even predictions on permuted biomass gives results which are better than random (median correlation is greater than 0). As expected, prediction is truly random when the correlation structure of the parental profiles is destroyed by permuting the cells of the parental profile matrix (Figure 3, Boxes J and K). Thus, the feature ranking found by predicting hybrid CRL class labels is of direct relevance to the prediction of average fresh weight.

## Discussion

In every plant, the genetic information is processed in a multitude of downstream processes (transcription, translation, post-translational modification, and metabolism) and in response to fluctuating environmental conditions, ultimately giving rise to a phenotype. For any given genome, the complete downstream process is highly complex and largely unknown, rendering the phenotype prediction based on genotype alone difficult. The combination of two parental genomes in a hybrid further leads to different levels of heterosis and adds yet another layer of complexity to the prediction problem. On the other hand, metabolic levels already integrate some of these processing steps (genetic predisposition and environmental conditions), are inexpensive to measure and have been shown to be closely connected to macroscopic traits such as biomass [39].

Here, we describe the analysis of the metabolic patterns of germinating roots of corn hybrids and their corresponding parental lines to ultimately predict HP. Little is known about the connection of parental and hybrid metabolite levels and all possible heterosis mode of action have been observed in the population under study [24]. We devised the concept of CRL which compares the hybrid level to each parent separately, thereby incorporating the diallel structure of the data. The discretization induced by the CRL also avoids potential non-linearity in hybrid metabolic levels.

We then classified the parental profiles with respect to the CRL labels for each metabolite, assuming that the outcome of each hybrid metabolite is influenced by the entire profile and not just its corresponding parental metabolite levels. We then aggregate the results of the separate classification problems to discover the parental metabolites which are most often influential in the hybrid outcome. To complete the chain of our model framework, we demonstrate that these same parental metabolites are more predictive of biomass than metabolites selected at random.

While each metabolite for each hybrid is compared to the corresponding parental metabolite levels in a univariate manner, it is not assumed that parental metabolites are determining hybrid levels independently of each other, but rather that the entire profile of both parents may be predictive. A simplified example is given in Figure S4. Presume that the level of metabolite X is low in genotypes A, B and C and high in D and E. If relationship between the average parental level and hybrid level is examined, it can be seen that even though both hybrids AxB and BxC have low average parental levels, the hybrid outcome is high and low, respectively. However, this disparity is in fact being driven by metabolite Y, whose average parental levels are, too, low and high, respectively. Furthermore, the interaction between parental metabolites may also be a function of level. For instance, when the average parental levels of DxE are high in X, this becomes the dominant influence, and causes the hybrid level to be moderate. This is despite the levels of Y being very close together in A, B, C and D.

To obtain a black-box predictor, we could have simply regressed parental profiles against root´s biomass and for added interpretability we could have used a purely statistical scheme for feature selection. However, this is a difficult task and ideally would require some form of validation. Additionally, prediction of biomass requires further optimization to choose the 'best' features, and it is not clear what criteria should be optimized. We circumvent this by choosing features that are highly relevant to metabolite shifts that have a solid biological interpretation, and validate them by demonstrating that they are additionally predictive of biomass.

We cannot claim that the predictive metabolites from this study are optimal predictors of biomass in experimental setups differing from ours. Additionally, the existence of multiple metabolic optima may confound a more straightforward prediction problem. Although it is not obvious how parental metabolic profiles influence HP and given that the genetic combining rules and the genetic-metabolic connections are unknown and likely to be complex, here, we demonstrate within one set of genotypes that the metabolic layer can be used as a proxy for the genetic layer, and by extension, that the parental profiles are predictive of biomass. Therefore, we have given a framework which can be easily applied to larger sets of genotypes.

The top ranked metabolites are postulated to have the greatest predictive power of fresh weight. However, it is not certain how robust the ranking is, or whether the most influential metabolites are interchangeable. From Figure 3 we can conclude that between 10 and 20 top metabolites are interchangeable, as beyond this point, inclusion of further metabolites decreases prediction accuracy. On the other end, regarding the bottom 50 metabolites, we see that these are rarely influential in predicting hybrid metabolite profiles and also have no predictive power for biomass. This suggests that either these metabolites do not act as a proxy for the genetic layer, or that it is encoded in a more complex manner than our model can capture. Amongst the top 5 metabolites are proline_m_, ketoglutaric acid_m_, histidine_m_ and trehalose_m_. In these cases always the maternal level (indicated by m) is more important in the prediction of hybrid outcome. This seems to be a general trend, as we find amongst the top 20 features only 5 paternal metabolite levels (Figure S5), and may be caused by both gene dosage effect in metabolite composition of kernel's triploid endosperm, the primary energy reserve as well as source for nourishment for a young corn seedling [40], [41] and the maternal inheritance of the plastidal genome in angiosperms. In accordance with our initial expectations regarding the usefulness of a feature selection, the hybrid class labels for histidine cannot be significantly predicted from parental levels, while the maternal level of histidine is highly predictive of the hybrids class labels of many other metabolites.

The present data set certainly is too small to allow more than speculative conclusions about these features. However, we have devised a conceptual framework of how genetic information may be processed when two genotypes are crossed, and attempted to apply machine learning methods to mimic such a process. The results of the described feature selection method might be better accessible to biological interpretation compared to black-box approaches. The results give a foundation for future investigation.

## Supporting Information

Figure S1 **(A)** **Idealized prediction workflow.** The aim of this study was to establish a mathematical framework, which allows to predict an integrative hybrid trait (Fresh Weight) from molecular parameters, namely levels of metabolites, obtained in the respective homozygous parents. **(B)** **Experimental setup and color scheme.** Root samples of four European maize lines and their twelve reciprocal hybrids were analyzed throughout this study.
(PDF)

Figure S2 **Class labels miss-classification frequency.** The median miss-classification frequency for class labels (indicating heterosis mode of action) of 69 metabolites showing balanced label sets obtained by SVM and compared against the minimum value obtained for permuted data sets (minperm). Metabolites are ordered according to the SVM misclassification rate for non-permuted data.
(PDF)

Figure S3 **Leave-one-out validation of feature ranking.** Feature ranking in a LOO approach compared to the original rank position of the parental metabolites. In general, ranking order is preserved, which potentially allows to apply the model to novel genotypes not included in the model building process.
(PDF)

Figure S4 **Independence of metabolite levels.** Metabolite levels cannot be regarded as independent from each other. In this example the hybrid level of metabolite X is dependent on the level of metabolite Y and can therefore not be predicted from the average parental value of X.
(PDF)

Figure S5 **Importance of maternal and paternal effects.** Parental metabolic features can be ranked according to their importance in hybrid class label prediction. Low ranks indicate metabolites which often important in prediction models. Maternal parental features are overrepresented among the top 20 metabolites from such a ranking. The Figure displays the number of maternal and paternal features up to a certain rank position. The further apart both lines are the stronger the effect is. At rank 20 for example we find 15 maternal and only 5 paternal metabolic features.
(PDF)

Figure S6 **Final model workflow.** Model workflow to perform a feature selection based on mid-parent heterosis, ultimately allowing to predict HP from parental metabolic profiles.
(PDF)

## References

[1] Duvick DN (2005) Genetic Progress In Yield Of United States Maize (Zea mays L.). Maydica 50: 193–202.

[2] Shull G (1914) Duplicate Genes for Capsule Form in Bursa bursa-pastoris. Z Indukt Abstamm Vererbungsl 12: 97–149.

[3] Zanoni U, Dudley JW (1989) Comparison of different methods of identifying inbreds useful for improving elite maize hybrids. Crop Sci 29: 577–582.

[4] Melchinger AE (1999) Genetic diversity and heterosis. In:Cors JG and Pandey S (eds) The Genetics and Exploitation of Heterosis in Crops. Crop Science Society of America, Madison, WI, 99–118.

[5] McWilliam JR, Griffing B (1965) Temperature-dependent heterosis in maize. Austral J Biol Sci 18: 569–583.

[6] Schrag TA, Möhring J, Melchinger AE, Kusterer B, Dhillon BS, et al (2010) Prediction of hybrid performance in maize using molecular markers and joint analyses of hybrids and parental inbreds. Theor Appl Genet 120: 451–461. 19916002 10.1007/s00122-009-1208-x

[7] Lenarcic AB, Svenson KL, Churchill GA, Valdar W (2012) A general Bayesian approach to analyzing diallel crosses of inbred strains. Genetics 190: 413–435. 22345610 10.1534/genetics.111.132563 PMC3276624

[8] Möhring J, Melchinger AE, Piepho HP (2011) REML-based Diallel Analysis. Crop Sci 51: 470–478.

[9] Cilas C, Bouharmont P, Boccara M, Eskes AB, Baradat P (1998) Prediction of genetic value for coffee production in Coffea arabica from a half-diallel with lines and hybrids. Euphytica 104: 49–59.

[10] Bernardo R (1996) Best Linear Unbiased Prediction of Maize Single-Cross Performance. Crop Sci 36: 50–56. 10.1007/BF00230131 24162487

[11] Vuylsteke M, Kuiper M, Stam P (2000) Chromosomal regions involved in hybrid performance and heterosis: their AFLP(R)-based identification and practical use in prediction models. Heredity 85: 208–218. 11012724 10.1046/j.1365-2540.2000.00747.x

[12] Maenhout S, De Baets B, Haesaert G, Van Bockstaele E (2007) Support vector machine regression for the prediction of maize hybrid performance. Theor Appl Genet 115: 1003–1013. 17849095 10.1007/s00122-007-0627-9

[13] Fu J, Falke KC, Thiemann A, Schrag TA, Melchinger AE, et al (2012) Partial least squares regression, support vector machine regression, and transcriptome-based distances for prediction of maize hybrid performance with gene expression data. Theor Appl Genet 124: 825–833. 22101908 10.1007/s00122-011-1747-9

[14] Yang W, Tempelman RJ (2012) A Bayesian antedependence model for whole genome prediction. Genetics 190: 1491–1501. 22135352 10.1534/genetics.111.131540 PMC3316658

[15] Schrag TA, Frisch M, Dhillon BS, Melchinger AE (2009) Marker-based prediction of hybrid performance in maize single-crosses involving doubled haploids. Maydica 54: 353–362.

[16] Technow F, Riedelsheimer C, Schrag TA, Melchinger AE (2012) Genomic prediction of hybrid performance in maize with models incorporating dominance and population specific marker effects. Theor Appl Genet 125: 1181–1194. 22733443 10.1007/s00122-012-1905-8

[17] Chen ZJ (2013) Genomic and epigenetic insights into the molecular bases of heterosis. Nat Rev Genet 14, 471–482. 10.1038/nrg3503 23752794

[18] Frisch M, Thiemann A, Fu J, Schrag TA, Scholten S, et al (2010) Transcriptome-based distance measures for grouping of germplasm and prediction of hybrid performance in maize. Theor Appl Genet 120: 441–450. 19911157 10.1007/s00122-009-1204-1

[19] Riedelsheimer C, Czedik-Eysenberg A, Grieder C, Lisec J, Technow F, et al (2012) Genomic and metabolic prediction of complex heterotic traits in hybrid maize. Nat Genet 44: 217–220. 22246502 10.1038/ng.1033

[20] Hoecker N, Keller B, Piepho HP, Hochholdinger F (2006) Manifestation of heterosis during early maize (Zea mays L.) root development. Theor Appl Genet 112: 421–429. 16362278 10.1007/s00122-005-0139-4

[21] Hoecker N, Keller B, Muthreich N, Chollet D, Descombes P, et al (2008) Comparison of maize (Zea mays L.) F1-hybrid and parental inbred line primary root transcriptomes suggests organ-specific patterns of non-additive gene expression and conserved expression trends. Genetics 179: 1275–1283. 18562640 10.1534/genetics.108.088278 PMC2475732

[22] Paschold A, Marcon C, Hoecker N, Hochholdinger F (2010) Molecular dissection of heterosis manifestation during early maize root development. Theor Appl Genet 120: 441–450. 19526205 10.1007/s00122-009-1082-6

[23] Hochholdinger F, Tuberosa R (2009) Genetic and genomic dissection of maize root development and architecture. Curr Opin Plant Biol 12: 172–177. 19157956 10.1016/j.pbi.2008.12.002

[24] Lisec J, Römisch-Margl L, Nikoloski Z, Piepho HP, Giavalisco P, et al (2011) Corn hybrids display lower metabolite variability and complex metabolite inheritance patterns. Plant J 68: 326–336. 21707803 10.1111/j.1365-313X.2011.04689.x

[25] Gärtner T, Steinfath M, Andorf S, Lisec J, Meyer RC, et al (2009) Improved Heterosis Prediction by Combining Information on DNA- and Metabolic Markers. PLoS ONE 4: e5220. 19370148 10.1371/journal.pone.0005220 PMC2666157

[26] Steinfath M, Gärtner T, Lisec J, Meyer RC, Altmann T, et al (2010) Prediction of hybrid biomass in Arabidopsis thaliana by selected parental SNP and metabolic markers. Theor Appl Genet 120: 239–247. 19911163 10.1007/s00122-009-1191-2 PMC2793375

[27] Cuadros-Inostroza A, Caldana C, Redestig H, Kusano M, Lisec J, et al (2009) TargetSearch - a Bioconductor package for the efficient preprocessing of GC-MS metabolite profiling data BMC Bioinform. 10: 428. 10.1186/1471-2105-10-428 PMC3087348 20015393

[28] Lisec J, Schauer N, Kopka J, Willmitzer L, Fernie AR (2006) Gas chromatography mass spectrometry-based metabolite profiling in plants. Nat Protoc 1: 387–396. 17406261 10.1038/nprot.2006.59

[29] Giavalisco P, Li Y, Matthes A, Eckhardt A, Hubberten HM, et al (2011) Elemental formula annotation of polar- and lipophilic-metabolites using (13) C, (15) N and (34) S isotope-labelling in combination with high-resolution mass spectrometry. Plant J 68: 364–376. 21699588 10.1111/j.1365-313X.2011.04682.x

[30] Kopka J, Schauer N, Krueger S, Birkemeyer C, Usadel B, et al (2005) GMD@CSB.DB: the Golm Metabolome Database. Bioinformatics. 21: 1635–1638. 10.1093/bioinformatics/bti236 15613389

[31] Smyth GK (2004) Linear models and empirical bayes methods for assessing differential expression in microarray experiments. Stat Appl Genet Mol Biol 3: Article3. 16646809 10.2202/1544-6115.1027

[32] Hastie T, Tibshirani R, Friedman JJH (2009) The Elements of Statistical Learning, Springer, 2nd. Ed.

[33] Breiman L (2001) Random Forests, Machine Learning. 45: 5–32.

[34] Boulesteix AL (2004) PLS dimension reduction for classification with microarray data. Stat Appl Genet Mol Biol 3: 33. 10.2202/1544-6115.1075 16646813

[35] Boulesteix AL, Strobl C (2009) Optimal classifier selection and negative bias in error rate estimation: an empirical study on high-dimensional prediction. BMC Med Res Methodol 9: 85. 20025773 10.1186/1471-2288-9-85 PMC2813849

[36] Slawski M, Daumer M, Boulesteix A-L (2008) CMA: a comprehensive Bioconductor package for supervised classification with high dimensional data. BMC Bioinformatics 9: 439. 18925941 10.1186/1471-2105-9-439 PMC2646186

[37] Fernie AR, Trethewey RN, Krotzky AJ, Willmitzer L (2004) Metabolite profiling: from diagnostics to systems biology. Nat Rev Mol Cell Biol 5: 763–769. 15340383 10.1038/nrm1451

[38] Sulpice R, Trenkamp S, Steinfath M, Usadel B, Gibon Y, et al (2010) Network analysis of enzyme activities and metabolite levels and their relationship to biomass in a large panel of Arabidopsis accessions. Plant Cell 22: 2872–2893. 20699391 10.1105/tpc.110.076653 PMC2947169

[39] Meyer RC, Steinfath M, Lisec J, Becher M, Witucka-Wall H, et al (2007) The metabolic signature related to high plant growth rate in Arabidopsis thaliana. Proc Natl Acad Sci U S A 104: 4759–4764. 17360597 10.1073/pnas.0609709104 PMC1810331

[40] Birchler JA, Yao H, Chudalayandi S (2007) Biological consequences of dosage dependent gene regulatory systems. Biochim Biophys Acta 1769: 422–428. 17276527 10.1016/j.bbaexp.2006.12.002 PMC1975783

[41] Guo M, Rupe MA, Danilevskaya ON, Yang X, Hu Z (2003) Genome-wide mRNA profiling reveals heterochronic allelic variation and a new imprinted gene in hybrid maize endosperm. Plant J 36: 30–44. 12974809 10.1046/j.1365-313x.2003.01852.x

