# Supplementary material for: Deducing Hybrid Performance from Parental Metabolic Profiles of Young Primary Roots of Maize by Using a Multivariate Diallel Approach
Source: PLoS One. 2014 Jan 7;9(1):e85435. doi: 10.1371/journal.pone.0085435 (PMC3883692; doi:10.1371/journal.pone.0085435)

Figure S1. (A) Idealized prediction workflow. The aim of this study was to establish a mathematical framework, which allows to predict an integrative hybrid trait (Fresh Weight) from molecular parameters, namely levels of metabolites, obtained in the respective homozygous parents.
(B) Experimental setup and color scheme. Root samples of four European maize lines and their twelve reciprocal hybrids were analyzed throughout this study.

A

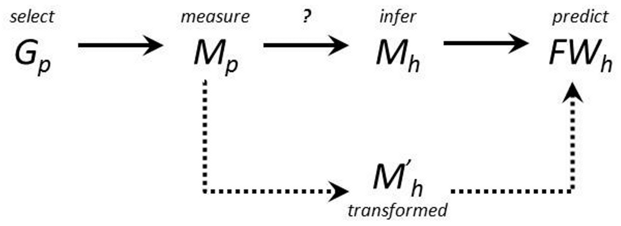

B

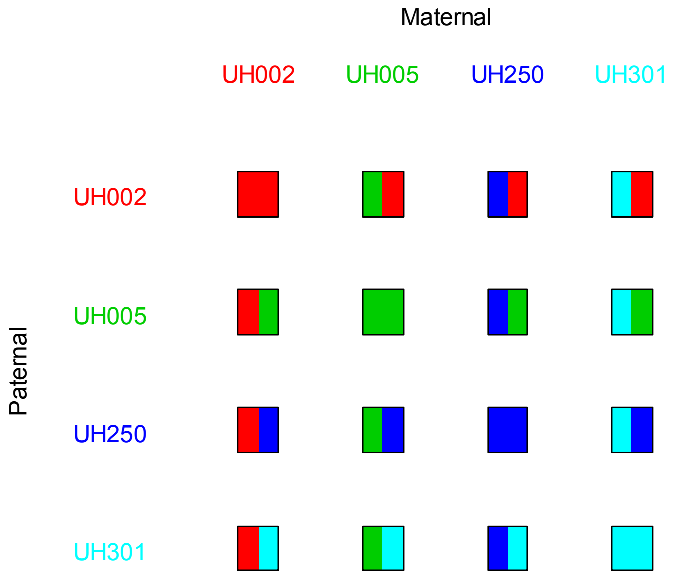

Supplement: Figure S1 — (A) Idealized prediction workflow. The aim of this study was to establish a mathematical framework, which allows to predict an integrative hybrid trait (Fresh Weight) from molecular parameters, namely levels of metabolites, obtained in the respective homozygous parents. (B) Experimental setup and color scheme. Root samples of four European maize lines and their twelve reciprocal hybrids were analyzed throughout this study. (PDF) [file pone.0085435.s001.pdf]
