# Supplementary figures and images for: Deducing Hybrid Performance from Parental Metabolic Profiles of Young Primary Roots of Maize by Using a Multivariate Diallel Approach
Source: PLoS One. 2014 Jan 7;9(1):e85435. doi: 10.1371/journal.pone.0085435 (PMC3883692; doi:10.1371/journal.pone.0085435)

Median Miss-Classification Frequency

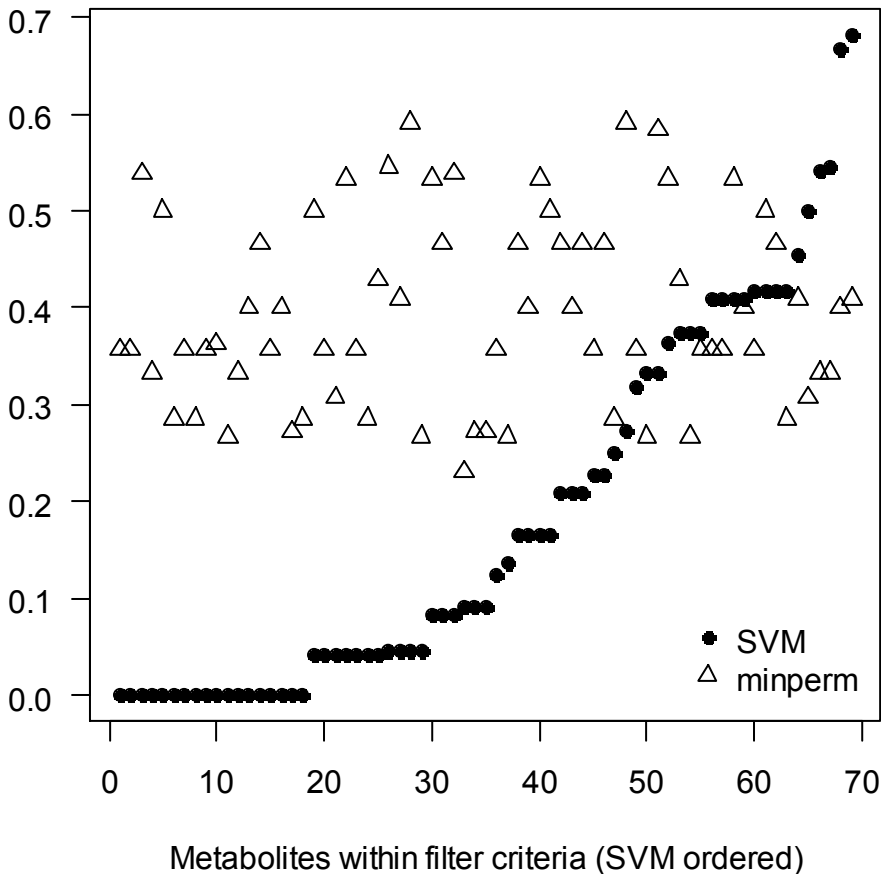

Supplement: Figure S2 — Class labels miss-classification frequency. The median miss-classification frequency for class labels (indicating heterosis mode of action) of 69 metabolites showing balanced label sets obtained by SVM and compared against the minimum value obtained for permuted data sets (minperm). Metabolites are ordered according to the SVM misclassification rate for non-permuted data. (PDF) [file pone.0085435.s002.pdf]

Median rank distribution from LOO-approach

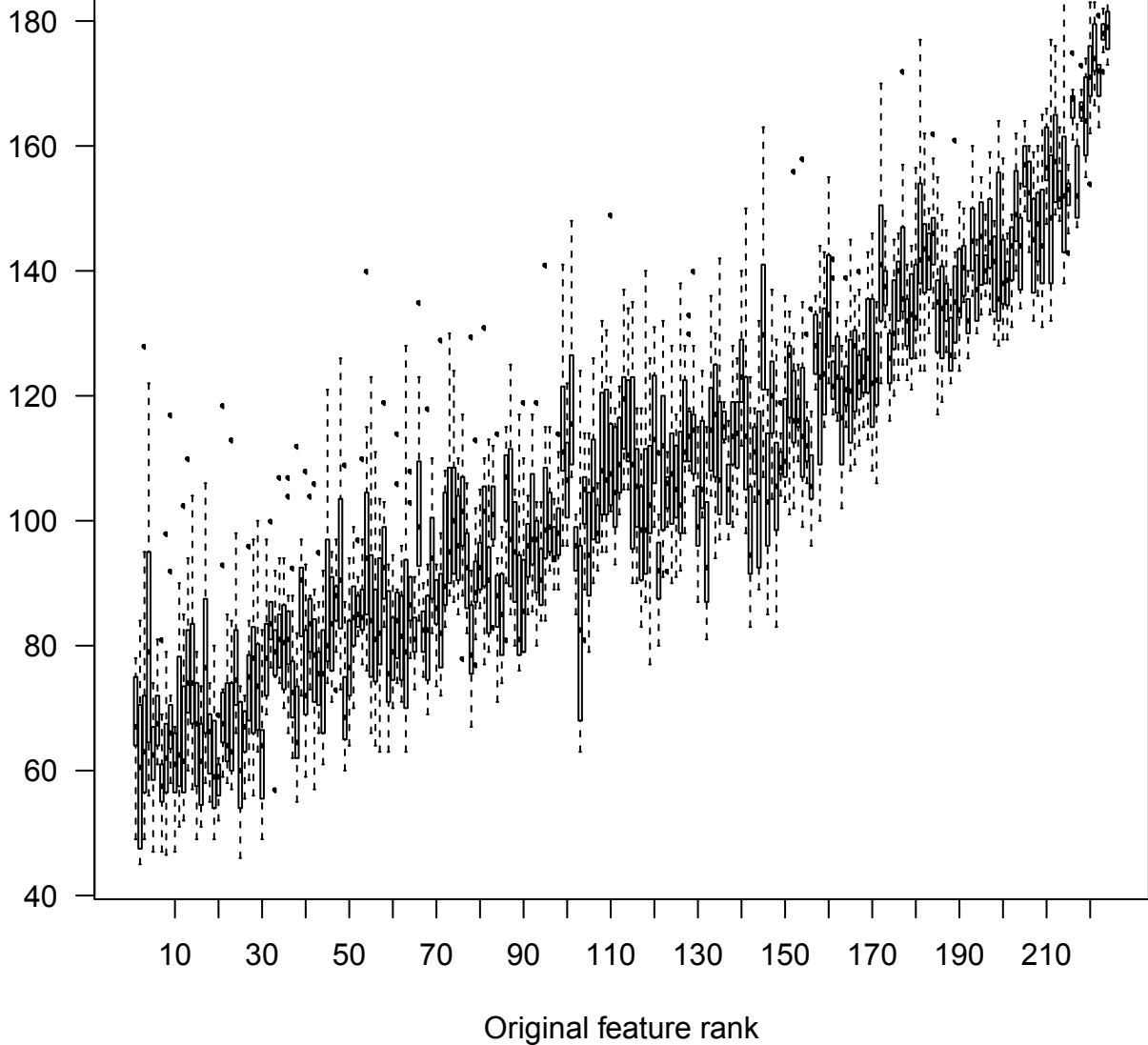

Supplement: Figure S3 — Leave-one-out validation of feature ranking. Feature ranking in a LOO approach compared to the original rank position of the parental metabolites. In general, ranking order is preserved, which potentially allows to apply the model to novel genotypes not included in the model building process. (PDF) [file pone.0085435.s003.pdf]

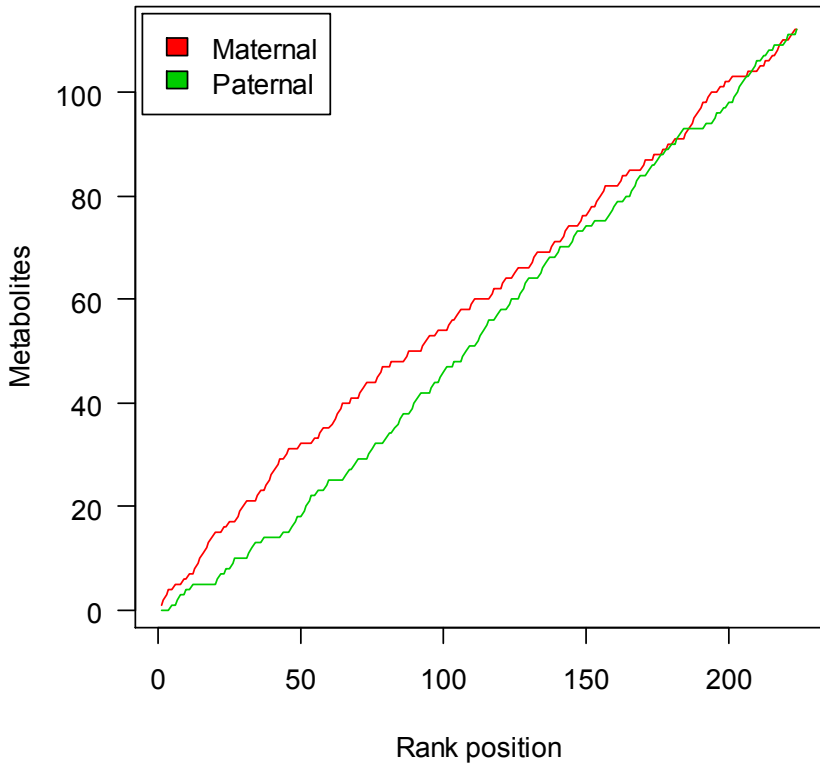

Supplement: Figure S5 — Importance of maternal and paternal effects. Parental metabolic features can be ranked according to their importance in hybrid class label prediction. Low ranks indicate metabolites which often important in prediction models. Maternal parental features are overrepresented among the top 20 metabolites from such a ranking. The Figure displays the number of maternal and paternal features up to a certain rank position. The further apart both lines are the stronger the effect is. At rank 20 for example we find 15 maternal and only 5 paternal metabolic features. (PDF) [file pone.0085435.s005.pdf]
