# Supplementary material for: Deducing Hybrid Performance from Parental Metabolic Profiles of Young Primary Roots of Maize by Using a Multivariate Diallel Approach
Source: PLoS One. 2014 Jan 7;9(1):e85435. doi: 10.1371/journal.pone.0085435 (PMC3883692; doi:10.1371/journal.pone.0085435)
Supplement: Figure S4 — Independence of metabolite levels. Metabolite levels cannot be regarded as independent from each other. In this example the hybrid level of metabolite X is dependent on the level of metabolite Y and can therefore not be predicted from the average parental value of X. (PDF) [file pone.0085435.s004.pdf]

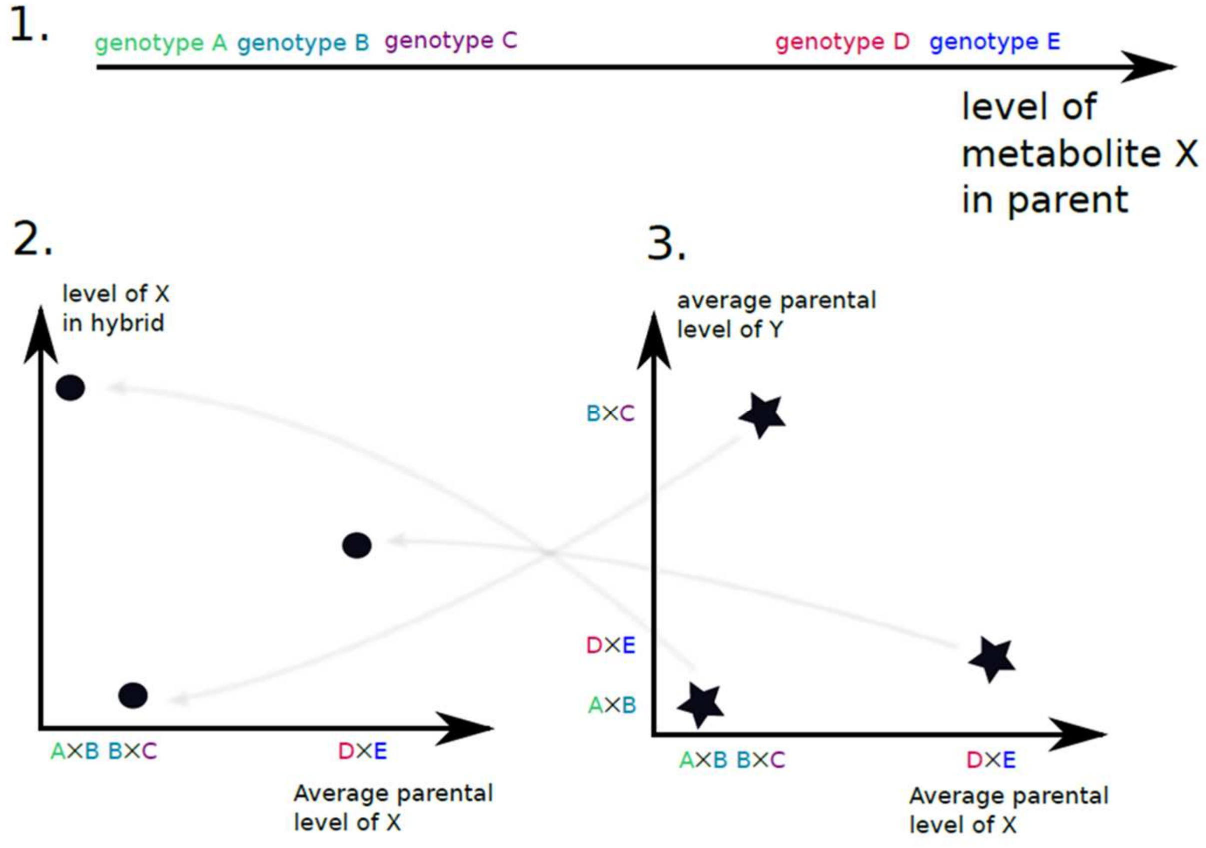
1.
genotype A genotype B genotype C
genotype D genotype E
level of metabolite X in parent
2.
level of X in hybrid
A×B B×C
D×E
Average parental level of X
3.
average parental level of Y
B×C
D×E
A×B
A×B B×C
D×E
Average parental level of X
