# Supplementary material for: Deducing Hybrid Performance from Parental Metabolic Profiles of Young Primary Roots of Maize by Using a Multivariate Diallel Approach
Source: PLoS One. 2014 Jan 7;9(1):e85435. doi: 10.1371/journal.pone.0085435 (PMC3883692; doi:10.1371/journal.pone.0085435)
Supplement: Figure S6 — Final model workflow. Model workflow to perform a feature selection based on mid-parent heterosis, ultimately allowing to predict HP from parental metabolic profiles. (PDF) [file pone.0085435.s006.pdf]

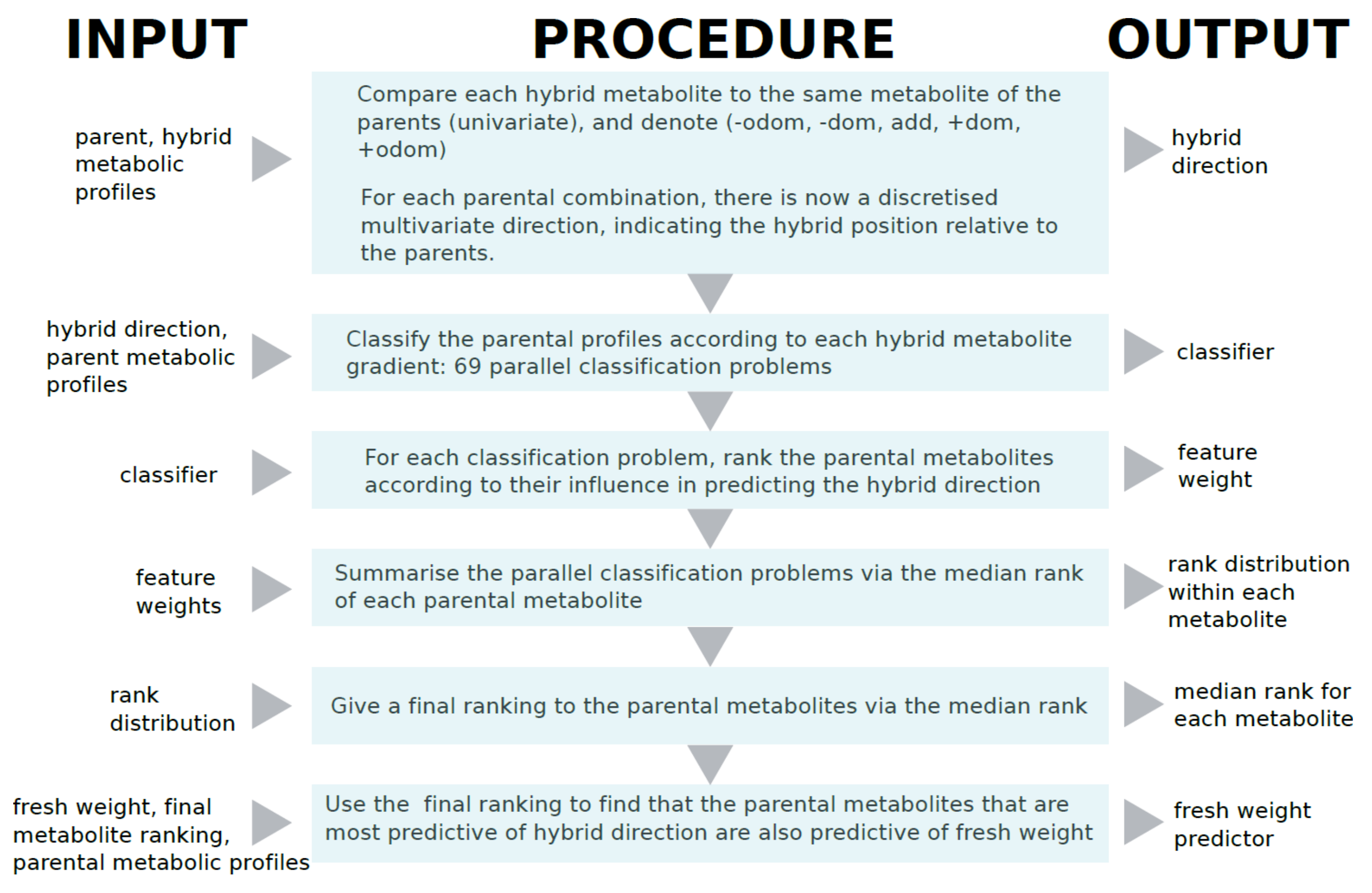
INPUT
PROCEDURE
OUTPUT
parent, hybrid metabolic profiles
Compare each hybrid metabolite to the same metabolite of the parents (univariate), and denote (-odom, -dom, add, +dom, +odom)
For each parental combination, there is now a discretised multivariate direction, indicating the hybrid position relative to the parents.
hybrid direction
hybrid direction, parent metabolic profiles
Classify the parental profiles according to each hybrid metabolite gradient: 69 parallel classification problems
classifier
classifier
For each classification problem, rank the parental metabolites according to their influence in predicting the hybrid direction
feature weight
feature weights
Summarise the parallel classification problems via the median rank of each parental metabolite
rank distribution within each metabolite
rank distribution
Give a final ranking to the parental metabolites via the median rank
median rank for each metabolite
fresh weight, final metabolite ranking, parental metabolic profiles
Use the final ranking to find that the parental metabolites that are most predictive of hybrid direction are also predictive of fresh weight
fresh weight predictor
